# Molecular and Morphometrical Characterization of *Fasciola* Species Isolated from Domestic Ruminants in Ardabil Province, Northwestern Iran

**Published:** 2017-03

**Authors:** Mojgan ARYAEIPOUR, Arezoo BOZORGOMID, Bahram KAZEMI, Masoud BEHNIA, Hakim AZIZI, Mohammad Bagher ROKNI

**Affiliations:** 1. Dept. of Medical Parasitology and Mycology, Shahid Beheshti University of Medical Sciences, Tehran, Iran; 2. Dept. of Medical Parasitology and Mycology, School of Public Health, Tehran University of Medical Sciences, Tehran, Iran; 3. Dept. of Biotechnology, School of Medicine, Shahid Beheshti University of Medical Sciences, Tehran, Iran; 4. Dept. of Medical Parasitology and Mycology, School of Medicine, Zabol University of Medical Sciences, Zabol, Iran

**Keywords:** *Fasciola hepatica*, *Fasciola gigantica*, morphology, Genotype, COI

## Abstract

**Background:**

We aimed to describe morphological and morphometrical characteristics of *Fasciola* spp. in livestock from Ardabil Province, Northwest Iran.

**Methods:**

Forty adult flukes were collected from different definitive hosts (cattle and sheep). Previously specimens were identified as *F. hepatica* or *F. gigantica* based on PCR-RFLP of the ITS-1 region with RsaI enzyme. We identified *Fasciola* spp. based on morphological and metric assessment of external features of fresh adults, morphological and metric assessment of internal anatomy of stained mounted worms. Statistical analysis was conducted using the Student’s t-test implemented in SPSS 15.0 (SPSS, Chicago, Illinois). Then the morphometric criteria of *Fasciola* samples were compared with PCR-RFLP data. The results of PCR-RFLP were confirmed by COI gene sequence.

**Results:**

The differences between the body length, area of the body, peripheral of the body, succer area, cone length, cone width, in two species were significant (*P* < 0.05). Based on Morphological characterizations, 6 specimens had the intermediate morphological features and 19 and 15 specimens had morphological features of *F. hepatica* and *F. gigantica*, respectively. In contrast, RFLP results showed, *F. hepatica* was present in 20 of the isolates, and *F. gigantica* in 20 isolates. No hybrid forms were detected.

**Conclusion:**

PCR-RFLP method can be used for differentiation of *Fasciola* species, which is more reliable method than morphology. Using morphology methods, merely, is not efficient for determination of genetic diversity.

## Introduction

Fascioliasis is primarily a disease of livestock such as cattle, sheep, and goats and humans are accidental hosts. This disease caused by liver flukes of the genus *Fasciola* (Platyhelminthes: Digenea: Fasciolidae). The life cycle is similar in both species and differs only in the intermediate host snail species. *F. hepatica* is mainly transmitted by *Galba truncatula*, widespread in temperate and subtropical climes. *F. gigantica* is transmitted by lymnaeids of *Radix auricularia* and *R. natalensis*, which live in the subtropics and tropics (1). Humans can acquire the infection by consuming water or raw aquatic plants contaminated with metacercariae (2).

Human fascioliasis in Iran is recognized by WHO as one of the six countries, known to have a serious problem (3). Both *F. hepatica* and *F. gigantica* has been prevalent in Iran and the infection rates of 0.1% to 91.4% have been reported in various livestock (4). Sheep are highly susceptible to fascioliasis caused by *F. hepatica*, and *F. gigantica* may be better adapted to cattle, with higher levels of resistance being observed in some breeds of sheep and goats (5).

The differentiation between *F. hepatica* and *F. gigantica* is important because both species are different in terms of epidemiological, control characteristics and pathogenicity. A recent study in Guirra sheep showed that, *F. hepatica* is less pathogenic than *F. gigantica*, because of the smaller size of *F. hepatica*, instead of genetic differences (6). These two species overlaps in many areas. Additionally, the presence of hybrid *Fasciola* forms have been reported in Gilan Province in north of Iran (7, 8). The presence of hybrid forms may have significant complications for people living in this province.

Despite the significant to distinguish between *Fasciola* species, the specific distinguishing can only be made by morphological study of adult flukes or molecular analysis (9). The microscopic analysis of samples is always recommended. These studies are rapid and inexpensive. Furthermore, such studies can be simply accomplished when a trained microscopist is present. Molecular technology has greater sensitivity than traditional methods, but at least now, their costs may prevent from their routine use.

Ardabil Province is located in Northwestern Iran, where fascioliasis is reported in both livestock and human. Anti-*Fasciola* antibodies were detected in sera of 1.96% of individuals by ELISA technique in this province (10). 25.9% of cattle, 5.3% of sheep, 11.4 % buffalo and 4.9% of goats were infected by *Fasciola* spp. (11).

In order to identify and describe the species of *Fasciola* occurring in livestock in this province, we carried out a study on slaughtered domestic animals, both with the traditional microscopic measurement and PCR-based technology. The presented article herein reports the morphometric characteristics of fasciolid adults in Ardabil Province, Northwestern Iran.

## Materials and Methods

### Study area

The present study was carried out in Ardabil, a province of Northwestern Iran (38.2514°N 48.2973°E). It is surrounded by Caspian Sea and the Republic of Azerbaijan. The province is known as one of the coldest provinces of Iran. It is very cold in winter and mild in summer. The average temperature annual is about seven degrees Celsius and the average annual rainfall in this region is about 500 mm.

### *Fasciola* Samples

Adult worms of *Fasciola spp*. were collected from the liver of 20 cattle and 20 sheep in Ardabil Province, Iran. Previously specimens were identified as *F. hepatica* or *F. gigantica* based on PCR-RFLP of the ITS-1 region with RsaI enzyme (12). The part presented herein is analyzing the morphometric characteristics of fasciolid adults. For morphometric analysis, the worms were washed in PBS and fixed between two slides and gentle pressure applied to flatten parasite, tie them with a cotton thin thread and for cleared keep the slid in lactophenol solution.

Species of helminths were identified based on their morphology and morphometry characters, using a microscope, equipped with camera lucida drawing tube. The morphological parameters were recorded in the questionnaire. Photography of the specimens was performed using a digital camera equipped microscopy. Morphological analysis was performed according to existing keys (body length (BL), body width (BW), body length to width ration (BL/BW); Cone Length (CL) etc) (4).

### DNA extraction and PCR

Genomic DNA was extracted from the adult flukes using (DNGTM-PLUS) kit (CinnaGen, Cat no. DN8117C, Iran) following manufacturer’s recommendations. The COI fragment was amplified by PCR (13), using a set of Ita8- (5′-ACGTTGGATCATAAGCGTGT-3)′ and Ita9-(5′-CCTCATCCAACATAACCTCT-3′) as forward and reverse primers, respectively.

For confirmation of morphometry results, PCR products of COI, of 6 isolates were sequenced on an ABI 3730XL capillary machine (Macrogen Inc., South Korea). Sequences data aligned and compared with those of existing sequences from the region, related to *Fasciola* spp. available in the GenBank, using the BLAST program of NCBI GenBank. Then nucleotide sequences of COI gene of the isolates obtained from the present study were deposited in the National Center for Biotechnology Information (NCBI) GenBank.

### Statistical Analysis

Student’s *t*-test was used to compare the mean of different variables between *F. hepatica* and *F. gigantica* and one-way analysis of variance (ANOVA) was used to determine whether there are any significant differences between the means of morphometric values in flukes isolated from different hosts.

Sequence results were edited and analyzed by Bio Edit software. The sequences were compared GenBank references sequences by BLAST program. Phylogenetic analysis predicated on COI sequence data were conducted using Neighbor-joining (NJ) method based on Tamura’s 3-parameter model using MEGA6. Bootstrap analysis was carried out with 1000 replications. DnaSP version 5.10.1 was used to determine the number of haplotypes and variable sites in each data set.

## Results

Forty adult *Fasciola* worms were collected from livers of 20 cattle and 20 sheep and slaughtered in Ardabil Province abattoirs, Iran during the period from December 2012 until October 2013.

### Morphological and morphometric characteristics:

Flukes with simple centripetal intestinal branches and small and club-shaped branches of the ovary were considered suggestive for *F. hepatica*. Those with complicated medial intestinal branches, further branching and branches of the ovary longer and more numerous with appearance that is more complicated were considered suggestive for *F. gigantica*. Furthermore, the mean length of *F. gigantica* was greater than *F. hepatica* and the less-developed shoulders.

The differences between the body length, area of the body, peripheral of the body, succer area, cone length, cone width, in two species were significant (*P* < 0.05). Based on morphological characterization and morphometric parameters specially BL/BW ratios in the present study the flukes were grouped into *F. hepatica*, *F. gigantica* and intermediate form. Six specimens had the intermediate morphological features and 19 and 15 specimens had morphological features of *F. hepatica* and *F. gigantica*, respectively. In contrast, RFLP results showed, *F. hepatica* was present in 20 of the isolates, and *F. gigantica* in 20 isolates. No hybrid forms were detected.

The morphometric characteristics of isolated flukes from sheep and cattle are summarized in Table 1.

**Table 1:** Comparative morphometrical data on adults of *F. hepatica and F. gigantica* from naturally infected cattle and sheep of the Ardabil Province, North West of Iran

**Adult measurements (mm)**	***F. gigantica* gravid adults cattle, Ardabil**	***F. hepatica* gravid adults sheep, Ardabil**
Body length, BL	19.14–40* (29.10± 5.51) **	13–31.5 (21.46±5.06)
Body width, BW	7–12.8 (10.14 ±1.9)	5–13.2 (9.48±2.74)
Cone length, CL	2.42–5.5 3.32 ±0.90	0.77–3.8 (2.40±0.69)
Cone width, CW	3–6.8 (4.47±1.24)	2.1–5 (3.20±0.66)
Oral sucker maximum diameter, OS max	0.66–1.1 (0.85±o.44)	0.33–0.88 (0.66±0.23)
Oral sucker minimum diameter, OS min	0.40–0.98 (0.56±0.097)	0.22–0.66 (0.43±0.16)
Ventral sucker maximum diameter, VS max	1.65–2.2 (1.85±0.18)	0.77–1.98 (1.38±0.53)
Ventral sucker minimum diameter, VS min	1.54–2.2 (1.74±0.22)	0.61–1.76 (1.08±0.23)
Distance between suckers, OS-VS	0.55–1.98 (1.32±0.25)	0.87–2.2 (1.35±0.44)
Distance between anterior end of body and VS, A-VS	1.8–2.53 (2.12±0.24)	1.32–2.9 (1.96±0.44)
Pharynx length (PhL)	0.24–0.88 0.66±0.17)	0.33–1 (0.64±0.1)
Pharynx width (PhW)	0.15–1.1 (0.51±0.24)	0.2–0.66 (0.38±0.13)
Testicular space length (TL)	7.35–15.3 12.57±2.51	5.32–25.5 (9.36±4.5)
Testicular space width (TW)	3.23–6.5 4.91±1.13	2.7–8 (5.23±1.69)
Body area (BA)	127.75–233.85 192.53±44.99	97.47–239 (161.12±75)
BL/BW ratio (BL/BW)	2.03–4.66 3.00±0.67	1.62–4 (2.38 ±0.67)
O.S area	0.22–0.86 0.54±0.15	0.1–0.45 0.3±0.08
V.S area	1.92–3.60 2.76±0.67	0.37–2.78 1.57±0.22
Sucker ratio OSA/VSA	0.11–0.23 0.17±0.24	0.27–0.81 0.54±0.15
Preipheral	95–51.83 75.85±15.09	35.53–91 56.87±25.61

* Minimum–maximum (mm).

** Mean ± SD.

### DNA Sequencing and Phylogenic Analysis

We used COI sequence for confirmation of results of morphometric characteristics. Based on having the highest BL/BW ratio, lowest BL/BW ratio, highest Sucker ratio, and lowest Sucker ratio, 7 samples were selected for sequencing. Partial sequences of COI of *F. hepatica* and *F. gigantica* showed 1 and 5 variable sites, respectively, and also yielded four haplotypes for *F. gigantica* and 2 haplotypes for *F. hepatica.* Sequences of COI of the isolates were deposited in the GenBank: accession numbers: KU605773 to KU605774 and KU682054 to KU682058 (Table 2). Phylogenetic analysis showed that the sequences KU682054 and KU682057 were similar to the Iranian *F. gigantica* sequence deposited in GenBank under the accession number GQ398050.1 Iran (100%), while KU682055 and KU682058 were similar to the Mauritanian *F. gigantica* sequence deposited in GenBank under the accession number HQ857104.1 (97%).

**Table 2:** Profile of *Fasciola* spp. sequenced in this study

**Specimen code**	**Host**	**Morphometric analysis**	**PCR-RFLP analysis**	**Sequence analysis**	**COI types Sequence Accession**	**Haplotype**
AD11	Sheep	*F.hepatica*	*F.hepatica*	*F.hepatica*	KU605773	H1
AD51	Cattle	*F.gigantica*	*F.gigantica*	*F.gigantica*	KU682056	G1
AD41	Cattle	*F.gigantica*	*F.gigantica*	*F.gigantica*	KU682054	G2
AD55	Cattle	*F.gigantica*	*F.gigantica*	*F.gigantica*	KU682057	G2
AD50	Cattle	*F.gigantica*	*F.gigantica*	*F.gigantica*	KU682055	G3
AD40	Cattle	*F.gigantica*	*F.gigantica*	*F.gigantica*	KU682058	G4
AD36	Sheep	*F.hepatica*	*F.hepatica*	*F.hepatica*	KU605774	H2

**Fig. 1:**
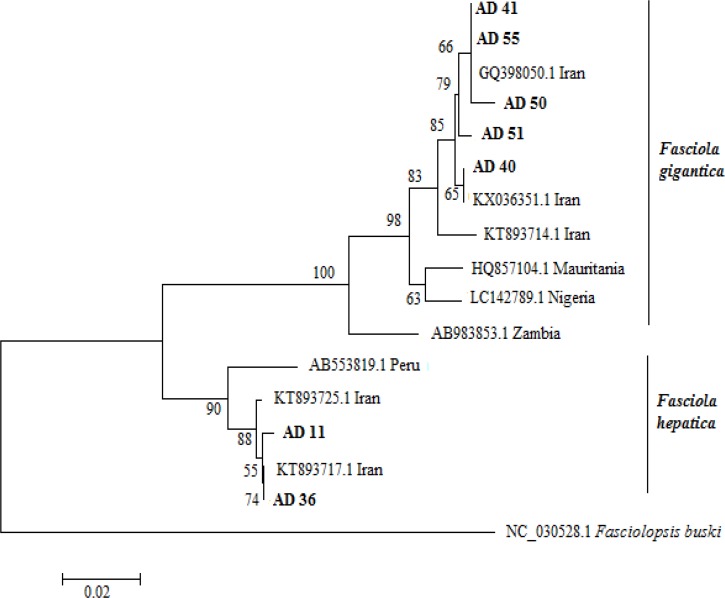
Neighbor-joining tree of COI nucleotide sequences (329 bp). Nodes are labeled with bootstrap values. Scale bars indicated nucleotide substitutions per site. Sequences obtained in this study are highlighted in bold font. The nucleotide sequence of the *Fasciolopsis buski* was used as outgroup

On the other hand, the sequences KU605773 and KU605774 were similar to the Iranian *F. hepatica* sequence deposited in GenBank under the accession number KT893717.1, 99% and 100% respectively (Fig. 1).

## Discussion

Fascioliasis is an important food-and water-borne parasitic zoonosis caused by *F. hepatica* and *F. gigantica*. The distribution of two species overlaps in most areas of world like Iran. Both *F. gigantica* and *F. hepatica* are transmitted in Ardabil Province. This makes it difficult to identify the particular species involved. Understanding the genetic identity of the parasite will be crucial for epidemiology, transmission dynamics characterizations and the control of such parasitic infections (14).

The emergence of various molecular approaches provided a sensitive and reliable tool for the identification and differentiation of helminthes parasites (15). Consequently, morphological tools have been undermined in recent years due to the loss of expertise and interest in traditional morphological studies. However, it is assumed that molecular and morphological characters are complementary in epidemiological studies on parasite zoonoses. Phenotypic studies had made major progress towards understanding inter- and intraspecific variation in *Fasciola* parasite leading to the identification of intermediate forms of the parasite.

Depending on previous studies the branching of the intestinal caeca, ovarian and testicular is an important aid for differentiation (16, 17). As to the cuticular armature, the shape and size of the scales were different in the two species (18). Accordingly, in the present study worms could be preliminarily grouped into *F. hepatica* and *F. gigantica*.

The distance between the ventral sucker and the posterior end of the body, body roundness and body length/body width ratio may be useful in discriminating *Fasciola* species (8, 19, 20). The measure of BL/BW has been considered as one of the useful indices for differentiating *F. hepatica* from *F. gigantica*. The BL/BW ratios of 1.29–2.80 and 3.40–6.78 are considered for *F. hepatica* and *F. gigantica*, respectively (19). In the present study, 19 and 11 specimens had the BL/BW values in the range of those reported for *F. hepatica* and *F. gigantica*, respectively, while 6 specimens showed having intermediate values between the two size ranges. Both *Fasciola* species and their intermediate form have phenotypically been characterized from Iran.

Ashrafi et al. has showed the presence of *F. hepatica, F. gigantica* and intermediate forms (*Fasciola* sp.) based the morphometric characteristics in Gilan and Mazandaran in Iran (8). In addition, using computer image analysis of morphometric characterization *F. hepatica* and *F. gigantica*, as well as of intermediate forms has been reported in Mazandaran Province, northern Iran (21).

Intermediate and several of morphological forms of *Fasciola* have been detected using traditional microscopic measurements in other countries such as Pakistan (22), Vietnam (23), Taiwan, the Philippines, Tunisia, Spain and Korea etc.

Due to the presence of morphometric differences and significant between the two species, means those morphometric measurements alone are not recommended for speciation. Therefore, in this study the *Fasciola* species was also characterized based on PCR-RFLP of the ITS-1 region with RsaI enzyme (12). Rsa1 bands under UV showed two different patterns for *F. hepatica* (367, 104, 68, 59, 54, and 28 bp size) and *F. gigantica* (367, 172, 59, 54, and 28 bp size). To confirm results PCR-RFLP, a fragment of 360 bp from the CO1 gene was sequenced for seven cases. In contrast with morphological results hybrid forms were not detected in the present study. Nevertheless, some researchers believe that employing morphological characteristics is useful in areas with the prevalence or low occurrence or no reported intermediate forms (24).

Our results agree with this finding that PCR-RFLP method can be used for differentiation of *Fasciola* species, which is more reliable method than morphology (9, 25).

The genetic diversity observed among the isolates in this study was not higher than that reported in previous studies, these results suggest that the population structure of *Fasciola* spp. in Ardabil Province are not fully differentiated from other regions of the country. However, a larger number of samples from this region would need to be collected and analyzed to confirm this idea.

As regards molecular of results, overall analysis showed low correlation between morphometric and genotyping. Molecular studies considered a powerful tool for differentiation between the two species of *Fasciola* even when the classical morphologic methods fail. Using morphology methods, merely, is not efficient for determination of genetic diversity. Characterization of the population genetic structures of *Fasciola* species is useful for the diagnosis and control of fascioliasis of humans and ruminants.

## Conclusion

PCR-RFLP method can be used for differentiation of *Fasciola* species, which is more reliable method than morphology.

## Ethical considerations

Ethical issues (Including plagiarism, informed consent, misconduct, data fabrication and/or falsification, double publication and/or submission, redundancy, etc.) have been completely observed by the authors.
